# Mechanism of Waterbird Diversity Succession and Its Contribution to Nutrient Loads in Chagan Lake, China

**DOI:** 10.1002/ece3.72583

**Published:** 2025-12-09

**Authors:** Xuemei Liu, Jingshuang Yang, Yanfeng Wu, Guangxin Zhang

**Affiliations:** ^1^ Northeast Institute of Geography and Agroecology Chinese Academy of Sciences Changchun China; ^2^ Jilin Chagan Lake National Nature Reserve Administration Songyuan Jilin China

**Keywords:** conservation strategies, driving mechanism, long‐term monitoring, nutrients load, waterbird diversity

## Abstract

Analyzing the spatiotemporal variation of waterbird diversity is important for wetland restoration and protection. However, further research is needed to investigate the mutual feedback between the mechanisms of waterbird diversity and water quality. Thus, we analyzed the waterbird diversity variations and their driving mechanisms in Chagan Lake from 2013 to 2022 based on piecewise structural equation modeling (SEM) and generalized additive model (GAM). The survey identified 197 waterbird species, 35 of which were endangered. The Anseriformes proportion has exceeded 50% over that period. Although the number and diversity of waterbird in the reserve have increased, they were also threatened by non‐ecological land (NEL) development and water quality deterioration. The NEL/EL, total nitrogen (TN), and total phosphorus (TP) significantly reduced waterbird diversity with coefficients of −0.66 (*p* < 0.001), −0.55 (*p* < 0.001), and −0.49 (*p* < 0.05), respectively. TN and TP loads of waterbird were 356,601 and 102,941 kg in 2022, respectively. Anseriformes and Gruiformes contributed the most total nitrogen and total phosphorus. The results identified waterbird as one of the exogenous sources of nutrient loads. Additionally, an appropriate NEL/EL threshold (< 1.2) was determined for protecting waterbird diversity. Three conservation strategies were proposed based on these findings. This study could provide theoretical support for waterbird protection in natural reserves.

## Graphical abstract

The population and biodiversity of waterbird in the reserve have increased. NEL and nutrient concentrations significantly increased TLI of lake and reduced waterbird biodiversity. Piscivorous waterbirds can significantly increase the phosphorus load and herbivorous waterbirds can increase nitrogen load. Waterbird biodiversity was more sensitive to the development and utilization of bareland and wetland. Waterbird biodiversity will significantly change when the NEL/EL ratio exceeded the threshold of 1.2.

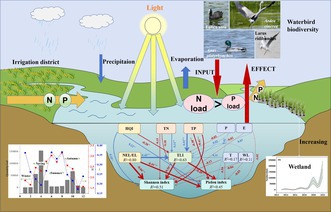

## 1 Introduction

Wetlands provide important habitats for migratory waterbirds and multiple ecosystem services despite covering a tiny fraction of the Earth's surface (Mitsch et al. 2015; Xu et al. 2020; Kačergytė et al. 2021). Over the past three centuries, an estimated 3.4 million km^2^ of inland wetlands have been lost worldwide, mainly due to agricultural expansions in regions such as the United States and China (Hu et al. 2017; Fluet‐Chouinard et al. 2023; Murray 2023). Rapid agricultural expansions caused water eutrophication by non‐point source pollution at higher latitudes and induced great changes in waterbird diversity (Wang et al. 2018; Hou et al. 2022). In order to effectively restore waterbird diversity and richness, numerous wetland restoration measures have been implemented worldwide (Kačergytė et al. 2021; Studholme et al. 2023). However, analyzing the driving pathways and contributions of multiple environmental factors to waterbird diversity was the prerequisite and foundation for formulating wetland restoration strategies.

The long‐term spatiotemporal dynamics and diversity variations of waterbird can reflect regional environmental changes (Furness and Greenwood 2013; Maznikova et al. 2024). In order to better reveal the driving pathways and contributions of multiple environmental factors to the dynamic variations of waterbird diversity, most studies focused on statistical models to simulate the pathways and relationships between variables (Xie et al. 2025; Gao et al. 2025). Previous studies on the influencing factors of waterbird diversity included hydro‐meteorology (e.g., temperature, precipitation, evaporation and water level), landscape pattern (e.g., urbanization and landscape fragmentation) and land use changes (Dowling et al. 2012; Żmihorski et al. 2016; Zuckerberg et al. 2018; Keten et al. 2020). Among them, precipitation and temperature were considered proximate predictors of waterbirds' community‐level responses to climatic changes (Pearce‐Higgins et al. 2015; Koshelev et al. 2021). Temperature increases altered waterbird community diversity and promoted their body size reduction (Şekercioğlu et al. 2012; Gaüzère et al. 2020; Neate‐Clegg et al. 2024). Higher landscape heterogeneity supported higher waterbird taxonomic diversity (Marcolin et al. 2023). Meanwhile, water levels affected the habitat suitability for migratory waterbird by altering vegetation distribution (Żmihorski et al. 2016; Zhu et al. 2021). Extensive studies focused on the relationship between environmental variables and waterbird species richness (Bacaro et al. 2011; Zellweger et al. 2016; Howard et al. 2020). How the distribution and driving mechanism of waterbird diversity in wetlands in high latitude agricultural gathering areas are key factors that constrain the balance between ecological protection and agricultural development. Hence, it is urgent to identify the pathways between multiple environmental factors and waterbird diversity for regional ecological governance and control.

Additionally, the interaction between water quality factors and waterbird diversity serves as the bond and key to wetland ecological protection (Singh and Prakash 2025; Zheng et al. 2025). Water quality factors such as algal toxins can directly cause the death of waterbirds, while pH, DO and nutrients indirectly reshape the distribution pattern of waterbirds by influencing osmotic pressure and the food chain (Rattner et al. 2022; Herbst 2023; Boros et al. 2025). Therefore, water quality serves as a “filter” for the distribution of waterbirds. Meanwhile, waterbirds have a bidirectional feedback effect on water quality through physiological metabolism and behavioral activities (Green and Elmberg 2014; Daebeler et al. 2020). On the one hand, a moderate and reasonable distribution of waterbirds can maintain ecological balance by preying on harmful organisms in the water (Li et al. 2023). On the other hand, high‐density feces intensify the input of exogenous nitrogen and phosphorus into water bodies, exacerbating eutrophication and the occurrence of algal blooms (Rodríguez‐Pérez and Green 2012; Fang et al. 2024; Abdel‐moneam et al. 2025). Hence, waterbirds are the “ecological indicators” and “dynamic regulators” of water quality. To enhance the effectiveness of regional ecological control, it is urgently necessary to further explore the interaction mechanism between water quality and waterbirds.

The Chagan Lake wetland, a typical national nature reserve in China, is selected to study the mechanisms underlying the interactions between water quality and waterbird diversity in the context of expanding irrigated saline‐alkali lands in recent decades. We hypothesized that waterbirds serve as nutrient sources for wetland water and are significantly affected by wetland water quality over the long term. To explore the bidirectional feedback mechanisms between waterbirds and water quality, (i) the spatiotemporal distribution and diversity of waterbird populations were investigated; (ii) the driving pathways and contributions of multiple environmental factors to the long‐term dynamic variations of waterbird diversity were revealed; (iii) and the contributions of waterbirds to the nutrient loads were quantified. Finally, on the basis of analyzing the feedback mechanism between waterbird diversity and multiple environmental factors, propose management strategies for ecological protection in nature reserves.

## 2 Material and Methods

### 2.1 Study Area

**FIGURE 1:**
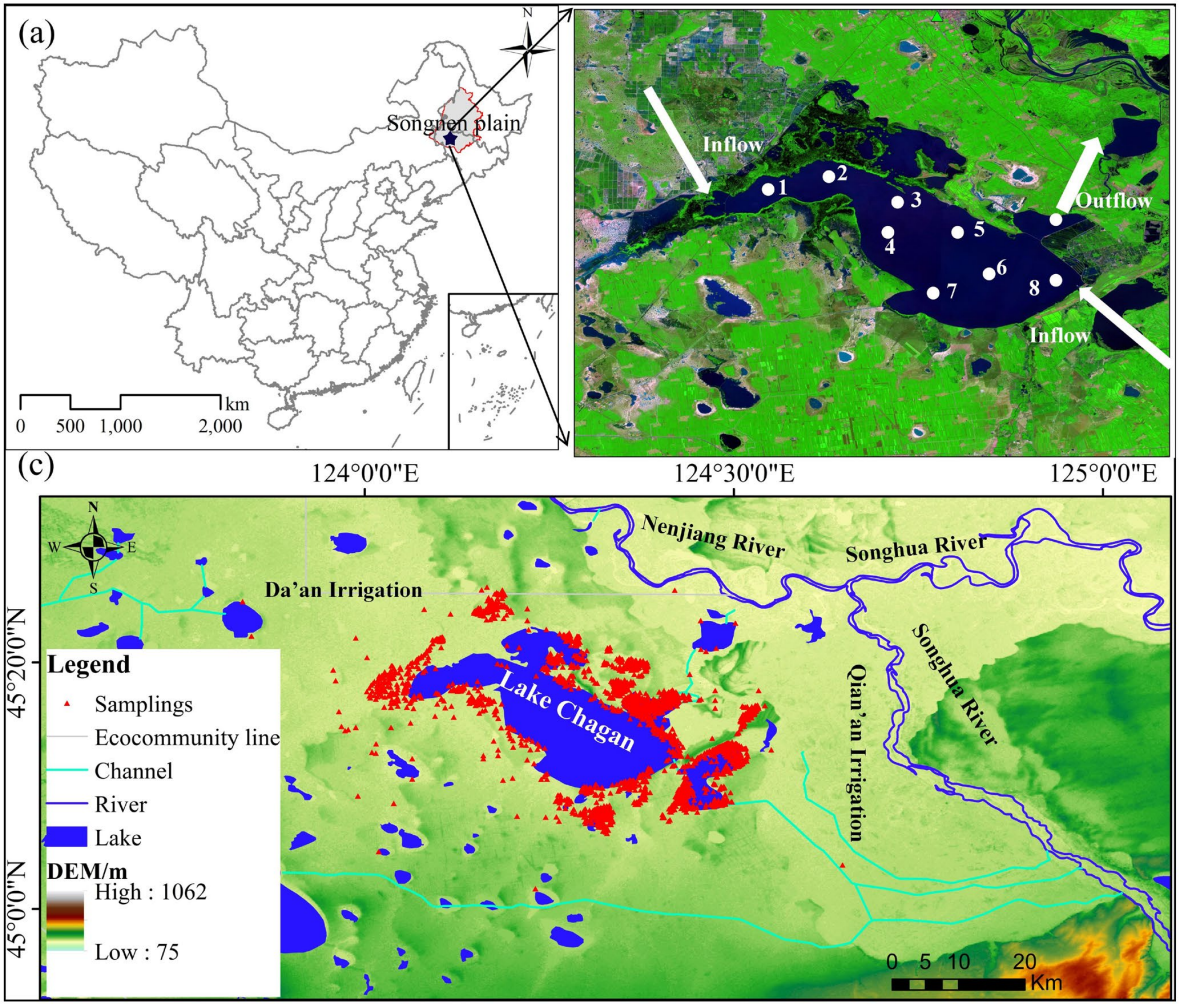
Study area and observation point distribution: (a) location of Chagan Lake wetland; (b) water quality sampling sites; (c) waterbird observation points.

Chagan Lake wetland is a typical seasonally ice‐covered national nature reserve for migratory waterbirds in Northeast China, renowned for winter fishing and hunting (Xu et al. 2024). Located in the southeastern region of the Songnen Plain, this wetland receives numerous irrigation discharges (Figure 1) as the primary input water source (Liu, Zhang, Xu, et al. 2020). As irrigation discharge transported salinity and nutrients into the wetland, water eutrophication was detected (Yang et al. 2015; Liu et al. 2019; Liu, Zhang, Zhang, et al. 2020) since a large amount of saline‐alkali lands and grasslands was transformed into farmlands (Liu et al. 2023). In recent years, degraded wetlands restoration and wetlands construction have restored the wetland area (Li et al. 2022; Liu et al. 2023).

### 2.2 Material

#### 2.2.1 Waterbird Survey

The abundance, species composition, and habitat of adult and juvenile waterbirds were investigated daily using binoculars during 2013–2022. To eliminate the error caused by inter‐investigator variability, each investigator was assigned fixed surveying tasks. Following the survey, a waterbird list with photographs was compiled. The list of waterbirds in the Chagan Lake National Wetland Nature Reserve was presented in Table S1.

#### 2.2.2 Water Quality Data Acquisition

Monthly (May to October) water quality data were obtained from nine observation stations during 2013–2022 (Figure 1b). Total nitrogen (TN), total phosphorus (TP), Chemical Oxygen Demand (COD_Mn_), pH, and salinity were analyzed at the Northeast Institute of Geography and Agroecology, Chinese Academy of Sciences. Annual variations in TN, TP, COD_Mn_, pH, and salinity from 2013 to 2022 are shown in Figure S1.

#### 2.2.3 Other Data

Landsat 5/8 multispectral data between 2013 and 2022 were collected from the United States Geological Survey (USGS) (https://earthexplorer.usgs.gov/). Annual land cover types were extracted using the GEE platform based on the spectral differences of various land cover types observed monthly (May to October). The changes in seven land cover types (e.g., wetland, paddy field, cornfield, water, bareland, grassland, and urban land) in the Chagan Lake basin between 2005 and 2022 are shown in Figure S2.

Hydrological (water level) and meteorological (temperature, precipitation, and evaporation) data were acquired from automatic weather stations and hydrographic stations. The water area data were extracted from remote‐sensing data. Annual hydrological and meteorological factor variations are presented in Figures S3 and S4.

### 2.3 Methods

#### 2.3.1 Analysis of Diversity

Based on field survey data and species distribution data, the waterbird species evenness index and diversity index were calculated (Equations 1 and 2).

(1) $$\text{Shannon index}\;\left(H\right):H=-\sum_{i=1}^{N}P_{i}\ln P_{i}$$

where *N* is the total number of waterbird species, and *P*_*i*_ is the proportion of the number of the *i*th species to the total number of waterbird. A greater *H* indicates greater diversity.

(2) $$\text{Pielou index}\;\left(J\right):J=H/\ln N$$

#### 2.3.2 Analysis of Land Use and Habitat Quality

The urbanization rate (Equation 3) is measured by the ratio of the urban population to the total population (including the urban and rural population) (Shi and Li 2018), which can be expressed as:

(3) $$\mathrm{UR}=\frac{\mathrm{UP}}{\mathrm{UP}+\mathrm{RP}}\times 100\%$$

where UR, UP, and RP represent the urbanization rate, urban population, and rural population, respectively.

The habitat Quality Index (HQI) is calculated as Equations (4) and (5):

(4) $$\mathrm{HQI}=A\times \left({\mathrm{WI}}_{i}\times {\text{HLand}}_{i}\right)/\text{Total Area}$$

(5) $${\text{HLand}}_{i}=\mathrm{SWI}\times {\text{Land}}_{i}$$

The weight index (WI) of each landscape type within the study area is shown in Table 1. The “*A*” indicates the normalization coefficient for the habitat quality index of aquatic wetland ecosystems, with a reference value of 785.60 (Shi et al. 2024).

**TABLE 1:** Weights and sub‐weights of landscape and sub‐landscape types of Chagan Lake Wetland.

Landscape types	Weight (WI)	Sub‐landscape types	Sub‐weight (SWI)
Grassland	0.23	Degraded grassland	0.1
Wetland	0.4	Lakes (channels)	0.3
		Tidal flats	0.3
Farmland	0.08	Paddy fields	0.6
		Cornfields	0.4
Construction land	0.01	Urban land	0.3
Unused land	0.1	Saline‐alkali land	0.3

The non‐ecological land (NEL) included bare land, urban land, and agricultural land. Ecological land (EL) included water, wetland and grassland. The higher the NEL/EL ratio, the higher the intensity of human development; while the lower the NEL/EL ratio, the lower the intensity of human development.

#### 2.3.3 Pathways and Threshold for the Response of Waterbird Diversity to Environmental Factors

The piecewise structural equation model (SEM) based on the principle of multivariate statistical analysis calculates the correlation and influence path of each variable, thereby analyzing multivariate causal relationships (Rabbetts et al. 2023; Yu et al. 2025). The pathways between waterbird community diversity and environmental factors can be explored using the constructed “piecewise SEM” R package. All possible paths have been identified and optimized. The correlation strength between two variables determines the significance (*p*) of the pathway. The simulation period for the model is selected from 2013 to 2022. The parameters that affect the diversity and evenness of waterbirds are divided into three levels. HQI and NEL/EL are considered the main variables that can highlight the impact of human activities on land use structure. TN and TP are the main water quality variables. Hydrological and meteorological variables include precipitation (*P*), evaporation (*E*), temperature (*T*), and water level (WL). Additionally, the generalized additive model (GAM) can balance the flexibility and interpretability of simulation (Viana and Chase 2022; Benito et al. 2022), and can effectively capture the nonlinear response tendency of waterbird diversity to various environmental factors. This process simulates the response of waterbird diversity to HQI, NEL and various land use types. The simulation results determined the threshold intervals of land use intensity that affect waterbird diversity.

#### 2.3.4 Nutrient Load of Waterbird

The calculation method for the annual contribution of waterbird to nutrient load is as follows Equations (6) and (7):

(6) $${\mathrm{NL}}_{N}=N_{i}\times 0.001\times F_{i}\times C_{N}\times D$$

(7) $${\mathrm{NL}}_{P}=N_{i}\times 0.001\times F_{i}\times C_{P}\times D$$

NL_N_ and NL_P_ indicate annual nutrient load (kg) of total nitrogen (TN) and total phosphorus (TP), respectively. *N*_*i*_ indicates the quantity of a certain type (*i*) of waterbird. *F*_*i*_ indicates daily dry matter excretion per individual (g/pcs/day). *C*_N_ and *C*_P_ indicate proportion of nitrogen and phosphorus in dry matter, respectively. *D* indicates the number of days waterbird were discovered within a year. NL_N_ and NL_P_ can calculate the contribution of waterbird to nutrient load in a certain habitat (land use type).

## 3 Results

### 3.1 Waterbird Population Composition and Distribution

#### 3.1.1 Community Composition

A total of 5679 waterbirds were observed and categorized into 19 orders, 44 families, 197 species. Among them, 10 were near‐threatened (NT) species, 16 were vulnerable (VU) species, 7 were endangered (EN) species, 2 were critically endangered (CR) species, and 162 were least concern (LC) species. In recent years, a critically endangered species, *Aythya baeri* , has also been frequently observed. In 2022, the number of adult *A. baeri* reached 31. Another critically endangered species, *Grus leucogeranus* , was occasionally observed. In the meantime, 13 national class I protected species and 31 national class II protected species were also observed (Table S1, Rodríguez et al. 2011).

**FIGURE 2:**
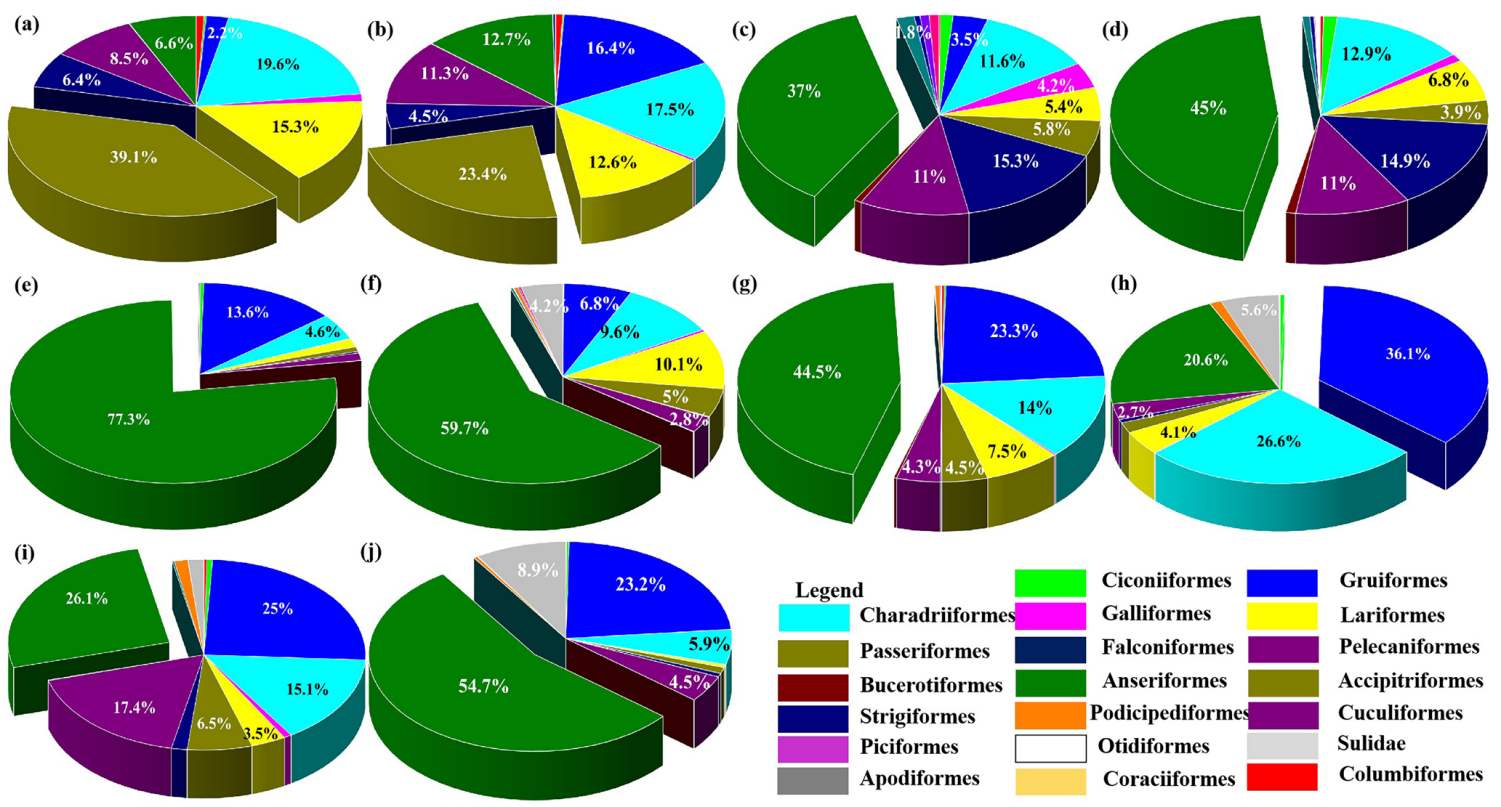
Proportions of waterbird orders from 2013 to 2022: (a–j) Proportions in each year from 2013 to 2022.

The proportions of waterbird orders changed greatly from 2013 to 2022 (Figure 2). The proportion of Passeriformes in the Chagan Lake Wetland National Nature Reserve showed a decreasing trend, while the proportions of Anseriformes and Gruiformes increased significantly. In recent years, the proportion of Anseriformes has exceeded 50%. In addition, Sulidae and Podicipediformes were recently observed in the reserve. Meanwhile, the proportion of Charadriformes fluctuated from 2013 to 2022.

#### 3.1.2 Spatial Differences of Waterbird

**FIGURE 3:**
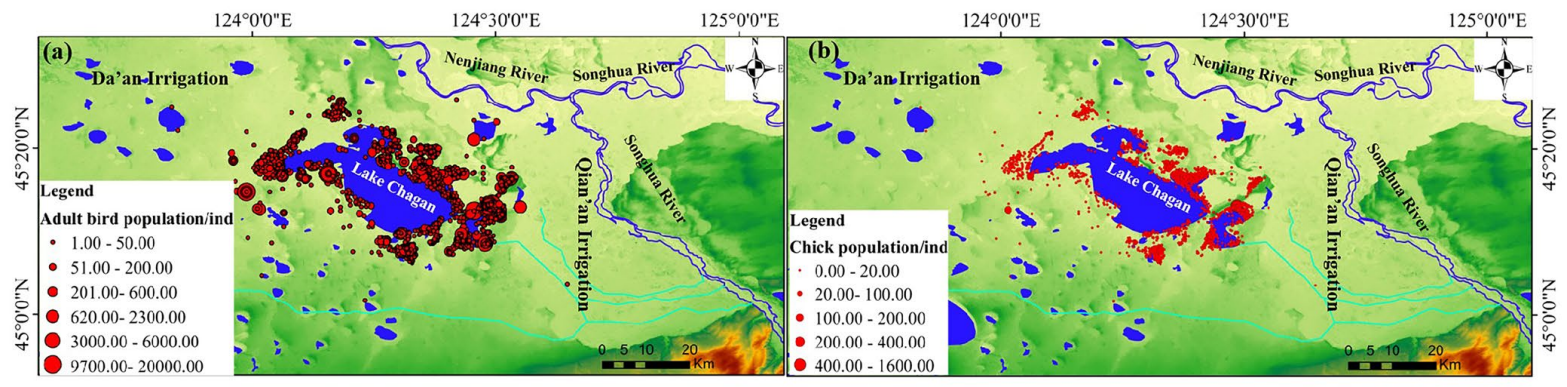
Spatial distribution of waterbird; Adult bird population distribution (a); Young bird population distribution (b).

The number of adult waterbirds was much greater than the number of juveniles (Figure 3). The adult waterbirds distributed densely around the wetland, while the juveniles distributed uniformly. Both adults and juvenile waterbirds were mostly distributed in shallow water or on the wetland. In contrast, the distribution of waterbirds in the NEL is rare.

#### 3.1.3 Temporal Differences of Waterbird

**FIGURE 4:**
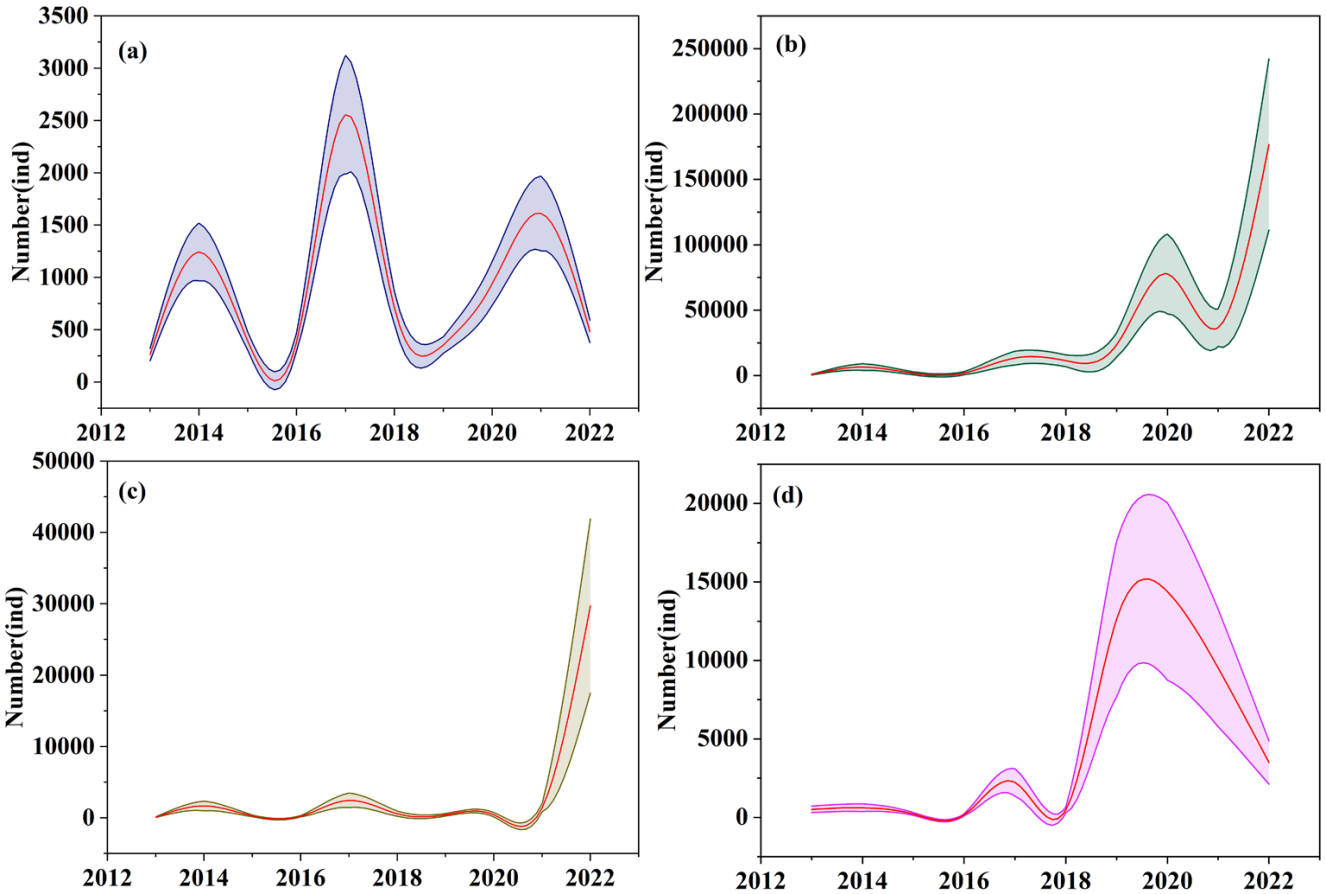
Annual variations of waterbird populations in different habitats: (a) Grassland; (b) Wetland; (c) Farmland; (d) In flight.

The number of waterbirds in different habitats tended to increase, especially in wetlands and farmland (Figure 4). The number of waterbirds in the grassland fluctuated significantly during 2013–2022. Over 150,000 waterbirds were spotted in wetlands, while nearly 30,000 were found on farmland. In addition, nearly 10,000 were found in flight.

**FIGURE 5:**
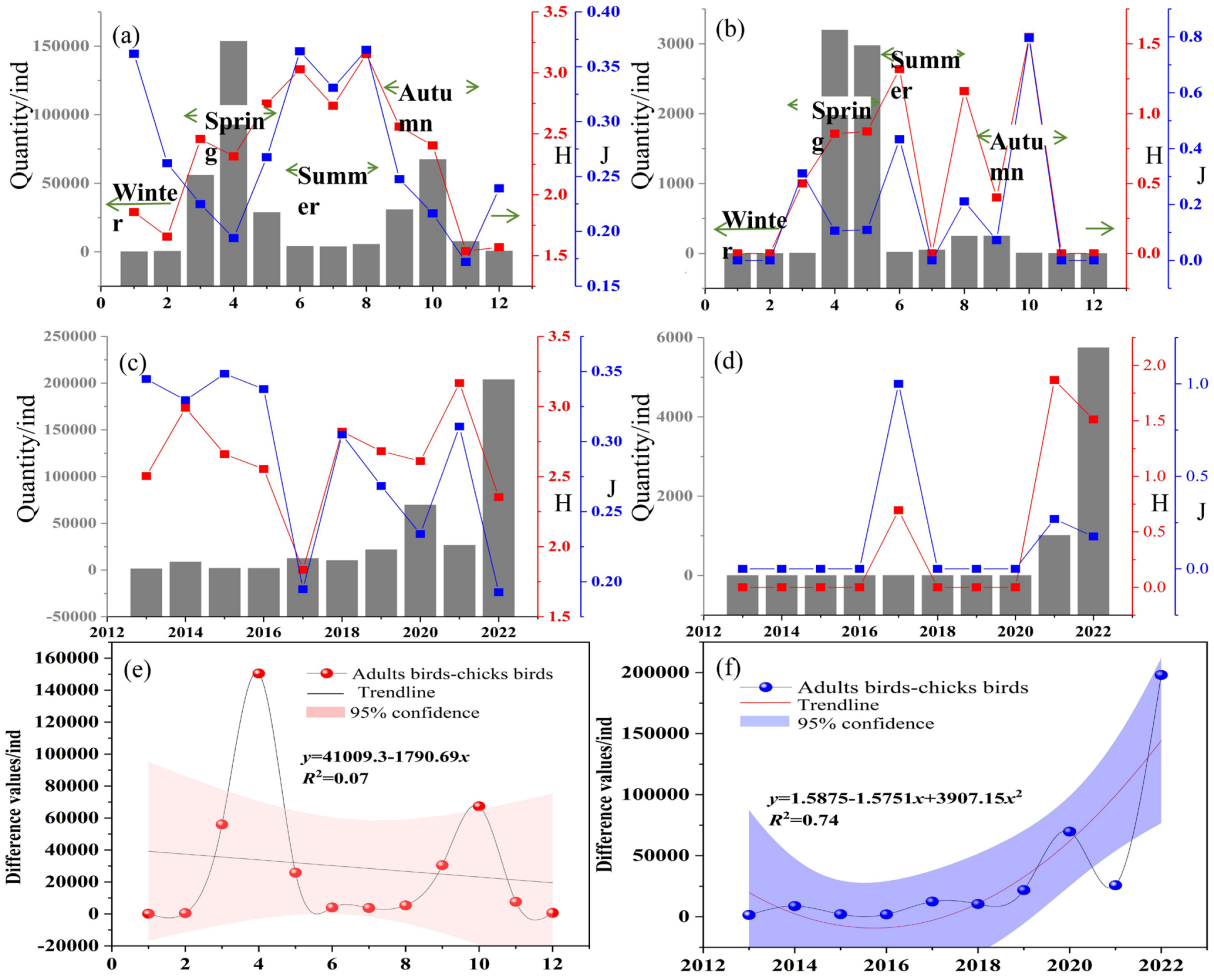
Temporal trends of the diversity and evenness of adult and juvenile waterbirds; (a) Monthly variations in adult waterbird diversity and evenness; (b) Monthly variations in juvenile waterbird diversity and evenness; (c) Annual variations in adult waterbird diversity and evenness; (d) Annual variations in juvenile waterbird diversity and evenness; (e) Monthly variations of waterbird reproductive rate; (f) Annual variations of waterbird reproductive rate.

The temporal trends of waterbird diversity and evenness in the Chagan Lake wetland exhibited obvious seasonality (Figure 5). The monthly variation of the adult waterbird population trended to be higher in spring and autumn but lower in summer and winter (Figure 5a). The peak monthly variations of adult waterbird diversity and evenness were observed in summer (Figure 5a). The monthly variations of the juvenile waterbird population trended to be higher only in spring (Figure 5b). However, the peak monthly variations in juvenile diversity and evenness were observed in autumn (Figure 5b). The diversity and evenness indexes of adult waterbird decreased slightly, while the diversity and evenness indexes of juvenile waterbird increased significantly (Figure 5c,d). The annual variations of the waterbird reproductive rate showed an increasing trend, and the monthly peak was observed in April (Figure 5e,f).

### 3.2 Response of Waterbird Diversity to Different Environmental Factors

#### 3.2.1 Waterbird Diversity

**FIGURE 6:**
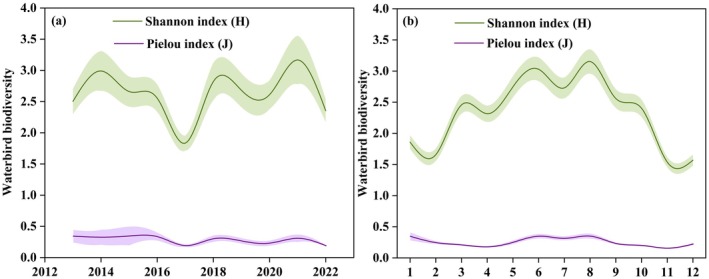
The temporal variations of waterbird diversity. (a) Annual variations, (b) monthly variations; Green and purple indicate the temporal variations of Shannon index (*H*) and Pielou index (*J*), respectively.

Shannon index (*H*) and Pielou index (*J*) showed consistency in temporal variation both on the annual scale and the monthly scale (Figure 6). The waterbird diversity showed a trend of first decreasing and then increasing during 2013–2022. The waterbird diversity reached a low point value in 2017. The seasonal variation of waterbird diversity was significant, reaching a peak value in summer.

#### 3.2.2 Identification of Waterbird Response Pathways to Environmental Factors

**FIGURE 7:**
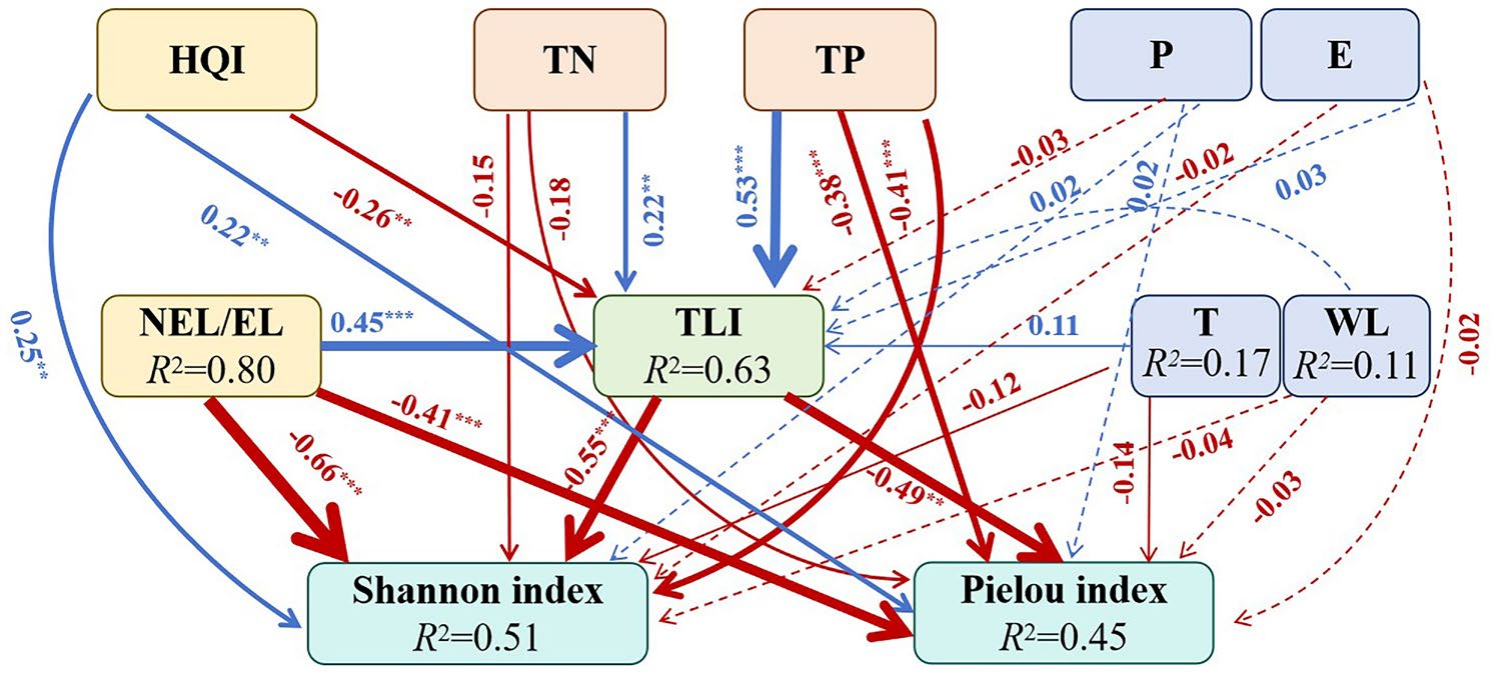
Response pathways of waterbird community diversity in wetland under the combined influence of human land use, nutrient load, and hydro‐meteorological factors. The numbers near the path arrow represent the standard path coefficients. **p ≤ 0.05, ***p ≤ 0.001. The blue and green lines indicate positive and negative impacts, respectively. Goodness‐of‐fit: AIC = 116.941, Fisher's *C* = 3.627, *p* = 0.182.

The impacts of different land use types on water quality and waterbird diversity were revealed by the piecewise SEM (Figure 7). This model explained the substantial variations in nutrient levels (TLI, *R* ^2^ = 0.63) and diversity (*H*, *R* ^2^ = 0.51; *J*, *R* ^2^ = 0.45), effectively capturing the mechanisms by which environmental factors affect waterbird communities. NEL (including bareland, urbanland and agricultural land) significantly increased TLI of lakes with coefficients of 0.45 (*p* < 0.001) and reduced waterbird diversity with coefficients of −0.66 (*p* < 0.001). Nutrient concentrations were the key water quality mediator between land use and waterbird diversity. TN and TP significantly increased TLI of lakes with coefficients of 0.22 (*p* < 0.05) and 0.53 (*p* < 0.001), respectively, and reduced waterbird diversity with coefficients of −0.55 (*p* < 0.001) and −0.49 (*p* < 0.05), respectively. Meanwhile, the impact of hydro‐meteorological factors on lake water quality and waterbird diversity was not significant. Therefore, land use changes significantly affected water quality and waterbird communities.

### 3.3 Nutrient Load Contributions of Waterbird

**FIGURE 8:**
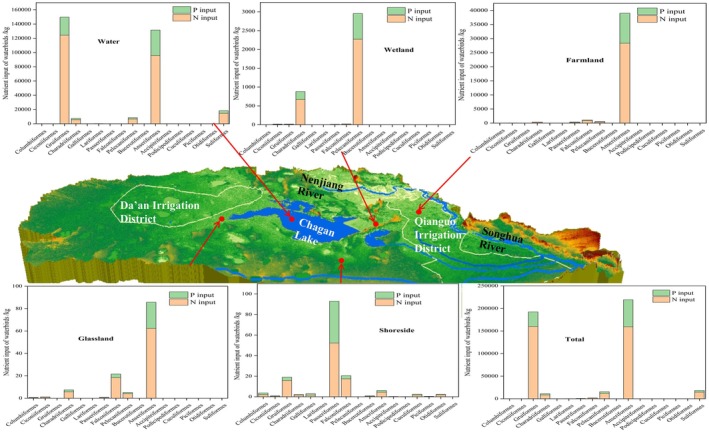
Contributions of different waterbirds to nutrient loads of their habitats in 2022.

The nutrient loads contributed by waterbirds in different habitats exhibited significant differences (Figure 8). The nutrient load contributions of waterbirds to water bodies, wetlands and farmland were greater than those to other habitats in 2022. The total nutrient loads of nitrogen and phosphorus were 356,601 and 102,941 kg in 2022, respectively. Among them, 69% of total nitrogen and 67% of total phosphorus were input into the water body, causing an increase in the nutrient status of the lake. Specifically, Anseriformes and Gruiformes contributed the most total nitrogen and total phosphorus. Charadriiformes and Pelecaniformes contributed significant total nitrogen to wetlands, while Ciconiiformes and Pelecaniformes tended to increase the phosphorus load. Anseriformes provided a certain amount of nitrogen and phosphorus to farmland, increasing the input of farmland nutrition. Passeriformes contributed significantly to the nitrogen and phosphorus load in grasslands. Falconiformes contributed significant nitrogen and phosphorus to shoreside. Overall, the nitrogen load input was greater than the phosphorus load input.

## 4 Discussion

### 4.1 Interaction Between Water Quality and Waterbird Diversity

The interaction between water quality and waterbird diversity was investigated. The results showed that nitrogen and phosphorus were the main water quality factors affecting waterbird diversity, while waterbirds were also the source of nutrient loads in water bodies, wetlands and farmland (Figures 7 and 8). Noteworthy, the water quality variations exhibited a negative feedback effect on waterbird diversity. These findings supported the previous claims that waterbirds increase the nutrient loads of water bodies (Figure 8; Tóth et al. 2023). Piscivorous waterbirds such as Pelecaniformes significantly increased the phosphorus load (Figure 8; Tóth et al. 2023). Meanwhile, herbivorous (e.g., Gruiformes) and omnivorous (e.g., Anseriformes) waterbirds increased the nitrogen load (Figure 8). Most studies have focused on the effects of toxic substances such as pesticides, petroleum, and plastic on waterbird diversity (Li et al. 2020; King et al. 2021; Wang et al. 2021). As a result, the effects of non‐point source pollution, such as nitrogen and phosphorus, on waterbird diversity were neglected, especially in areas with farmland aggregation. Excessive nitrogen and phosphorus nutrients can cause eutrophication and algal blooms (Huisman et al. 2018; Brookfield et al. 2021; Song et al. 2023). Deteriorated water bodies directly caused waterbird deaths through the food chain or indirectly reduced the waterbird population by changing their habitat environment (Rattner et al. 2022; Gladyshev et al. 2023). Most of the existing studies focused on the effects of algal blooms on the stress levels and immune functioning of waterbirds (Refsnider et al. 2021; Rattner et al. 2022; Demertzioglou et al. 2022), and few studied the effects of various water quality indicators on the population and diversity of waterbirds. This study showed that TN and TP significantly increased TLI of the lake and reduced waterbird diversity (Figure 7). These results were also influenced by the changes in human land use types. Excess nutrients can cause water quality deterioration and habitat destruction, resulting in a decline in waterbird diversity (Sullivan et al. 2021; Peneaux et al. 2021). The monthly nutrient variations promoted vegetation growth, which was conducive to the restoration of wetlands and other habitats (Jiang et al. 2021; Walton et al. 2020). Hence, the annual water quality variations had a negative feedback effect on waterbird diversity.

### 4.2 Variations in Waterbird Diversityat Different Land Use Intensities

**FIGURE 9:**
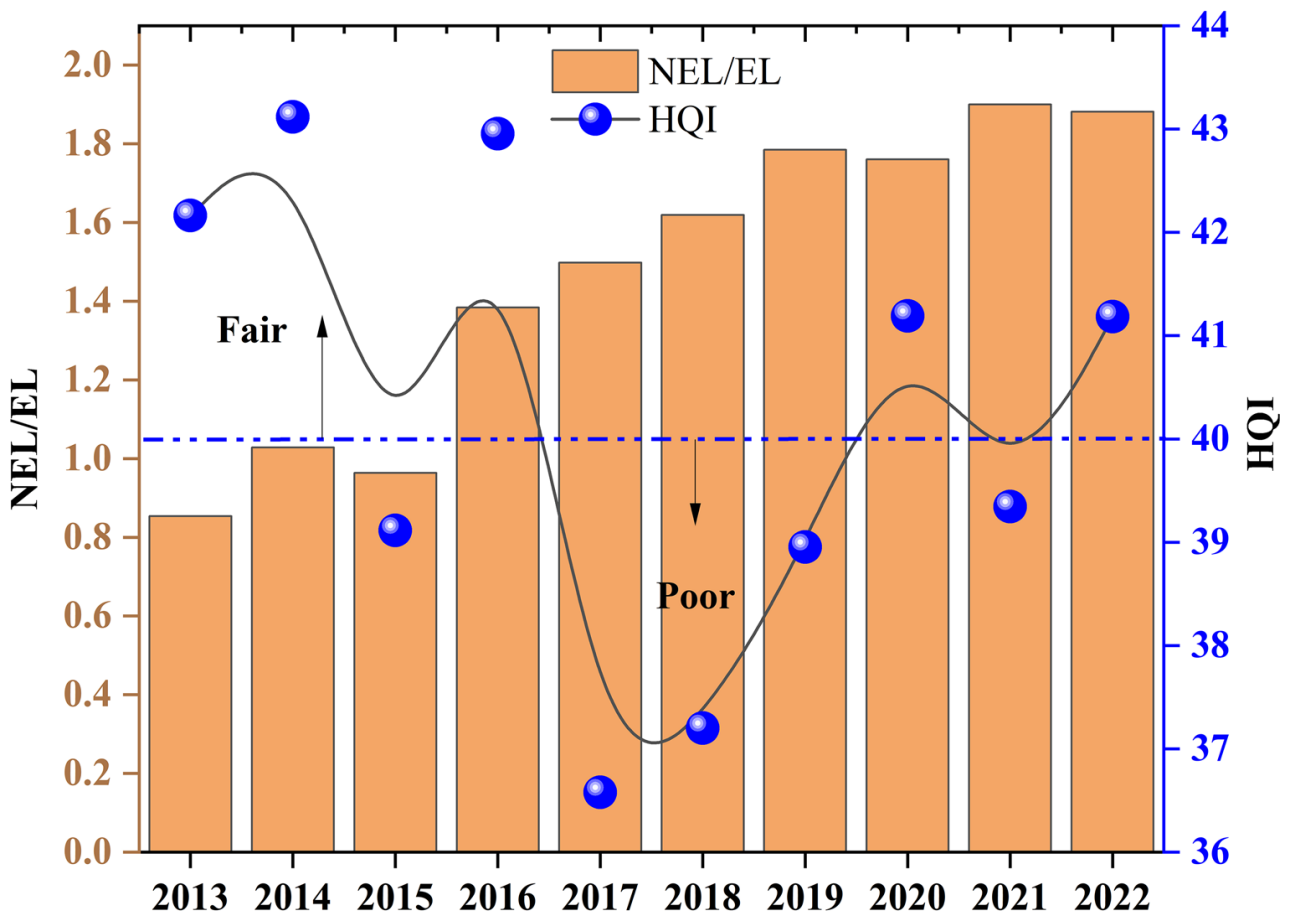
Annual HQI and NEL/EL variation characteristics from 2013 to 2022.

Land use was an important link between human activities and the ecological environment, driving changes in waterbird distribution and diversity by affecting habitat quality (Wretenberg et al. 2010; Allen et al. 2019; Tu et al. 2020). In the Chagan Lake Wetland National Nature Reserve, most of the grassland has been transformed into farmland (Figure S6). The farmland expansion and wetland restoration led to an increasing trend in waterbird populations on farmlands and wetlands (Figure 4). Hence, the land use changes caused significant changes in waterbird distribution. Habitat Quality Index (HQI) was used to evaluate the status of waterbird habitats within protected areas. NEL/EL ratio was also used to evaluate the degree of human land use and development. The waterbird habitat quality in Chagan Lake wetland showed a V‐shaped variation pattern (Figure 9). Overall, the HQI of the Chagan Lake wetland was about 40, which indicates a relatively fragile habitat. The NEL in Chagan Lake wetland was increasing intendency during 2013–2022, threatening the area of waterbird habitats (Figure 9). Different levels of land use intensity can drive changes in waterbird diversity (Rabbetts et al. 2023). Determining appropriate land use thresholds was crucial for balancing waterbird management and land use economic development in wetland conservation (Yu et al. 2025).

**FIGURE 10:**
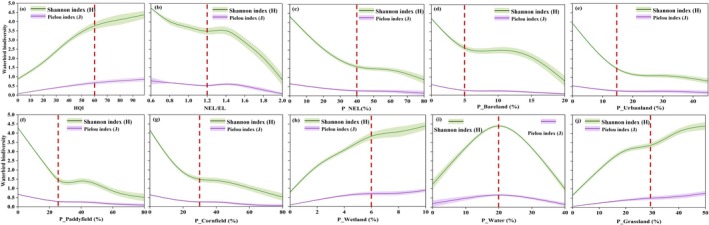
Response thresholds of waterbird diversity under different land use intensity.

The waterbird diversity conditions will change significantly when NEL/EL is between 0.6 and 2 (Figure 10). When the NEL/EL exceeds the threshold of 1.2, significant changes will occur in the waterbird population in Chagan Lake wetland. It is worth noting that waterbird diversity displays a higher sensitivity to bareland and wetland use compared to paddyfield and cornfield, with response thresholds ranging from 5% to 10% for bareland, 4%–6% for wetland and 25%–40% for paddyfield, 30%–40% for cornfield (Figure 10). However, the non‐point source pollution caused by agricultural land cannot be ignored with the construction of regional grain production projects (Shen et al. 2012; Liu et al. 2013; Zou et al. 2020). Those excess nutrient loads threaten the survival of waterbirds (Johnson et al. 2010; Manning and Sullivan 2021; Richard et al. 2021).

### 4.3 Wetland Waterbird Conservation Strategies

The reserve has a mixed impact on waterbirds, requiring targeted management (Wauchope et al. 2022). Our findings could inspire targeted conservation strategies.

1. The reserve should strengthen the protection of shallow water bubbles around the Chagan Lake based on the spatial distribution characteristics of waterbird.
2. More attention should be paid to the impact of bareland development and wetland destruction on waterbird diversity in the Chagan Lake National Nature Reserve. The ratio of NEL/EL should not exceed 1.2.
3. Water quality control targets should be more strictly designed to take into account the input of nutrients into water bodies by waterbird, especially the control of total nitrogen.

## 5 Conclusion

Based on the population, species, and habitats of waterbirds during 2013–2022, the diversity variations and their driving mechanism in the Chagan Lake Wetland National Nature Reserve were analyzed. Although the number and diversity of waterbirds in the reserve have increased, they were also threatened by NEL development and water quality deterioration. Noteworthy, NEL and nutrient concentrations significantly increased TLI of the lake and reduced waterbird diversity. The impact of hydro meteorological factors on waterbird diversity was not significant. The input of TN by waterbirds to the reserve was greater than that of total phosphorus. Among them, 69% of total nitrogen and 67% of total phosphorus were input into the water body, causing water quality deterioration of the lake. Notably, Anseriformes and Gruiformes contributed the most total nitrogen and total phosphorus. Additionally, waterbird diversity was more sensitive to the development and utilization of bareland and wetland. The waterbird diversity in Chagan Lake wetland will significantly change when the NEL/EL ratio exceeds the threshold of 1.2. Based on these findings, three suggestions were proposed concerning waterbird protection for sustainable development.

## Author Contributions

**Xuemei Liu:** conceptualization (lead), data curation (equal), formal analysis (lead), funding acquisition (lead), methodology (lead), software (lead), visualization (lead), writing – original draft (lead), writing – review and editing (equal). **Jingshuang Yang:** data curation (equal), formal analysis (equal), investigation (lead). **Yanfeng Wu:** funding acquisition (equal), supervision (equal), writing – review and editing (equal). **Guangxin Zhang:** writing – review and editing (equal).

## Funding

This research was supported by the National Natural Science Foundation of China (Grant Nos. 42207088, U23A2008, and 42571039); National Natural Science Foundation of Jilin Province (YDZJ202401475ZYTS).

## Conflicts of Interest

The authors declare no conflicts of interest.

## Supporting information


**Figures S1–S6:** ece372583‐sup‐0001‐FigureS1‐S6.docx.

**Table S1:** ece372583‐sup‐0002‐TableS1.docx.

## Data Availability

All the required data is uploaded as Supporting Information.
