# Supplementary material for: Mechanism of Waterbird Diversity Succession and Its Contribution to Nutrient Loads in Chagan Lake, China
Source: Ecol Evol. 2025 Dec 9;15(12):e72583. doi: 10.1002/ece3.72583 (PMC12687063; doi:10.1002/ece3.72583)
Supplement: Supplementary file 1 — Figures S1–S6: ece372583‐sup‐0001‐FigureS1‐S6.docx. [file ECE3-15-e72583-s002.docx]

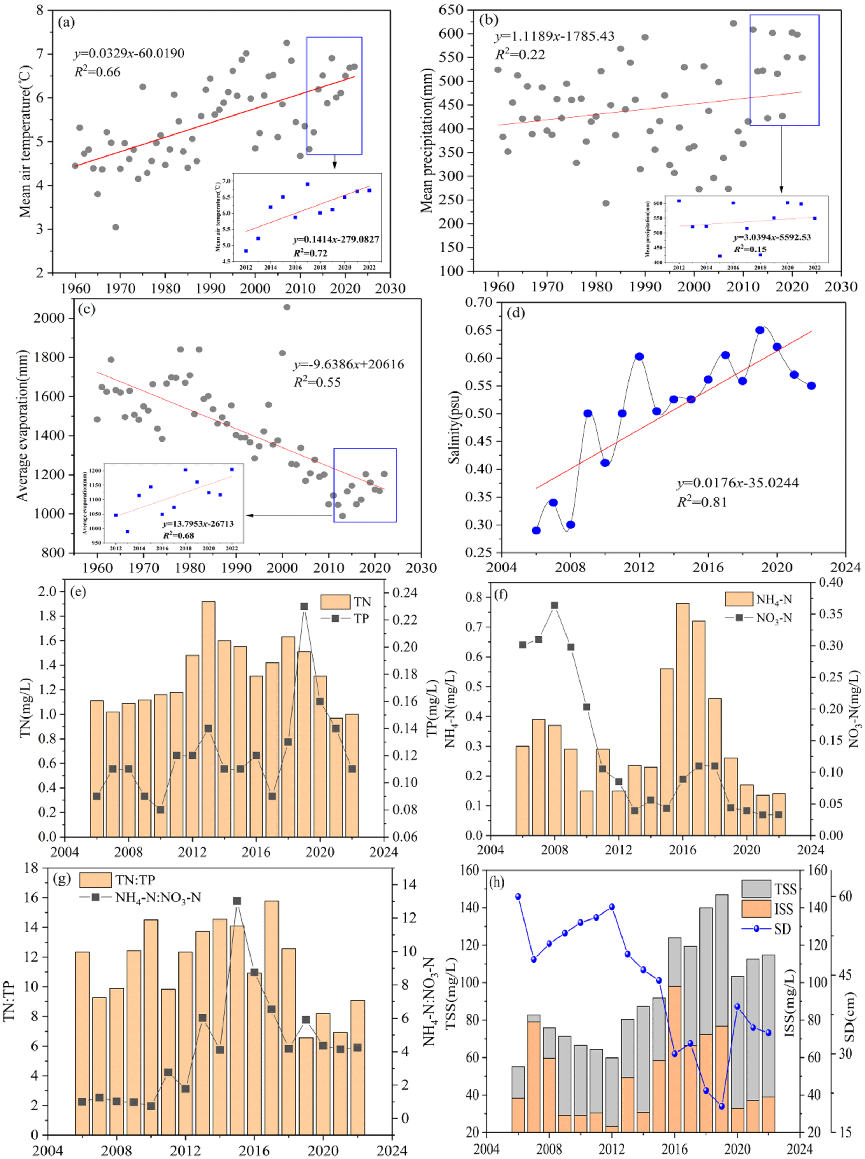


Figure S1. Annual variation of total nitrogen (TN), total phosphorus (TP), Chemical Oxygen Demand (COD_Mn_), pH, and salinity during 2013-2022.


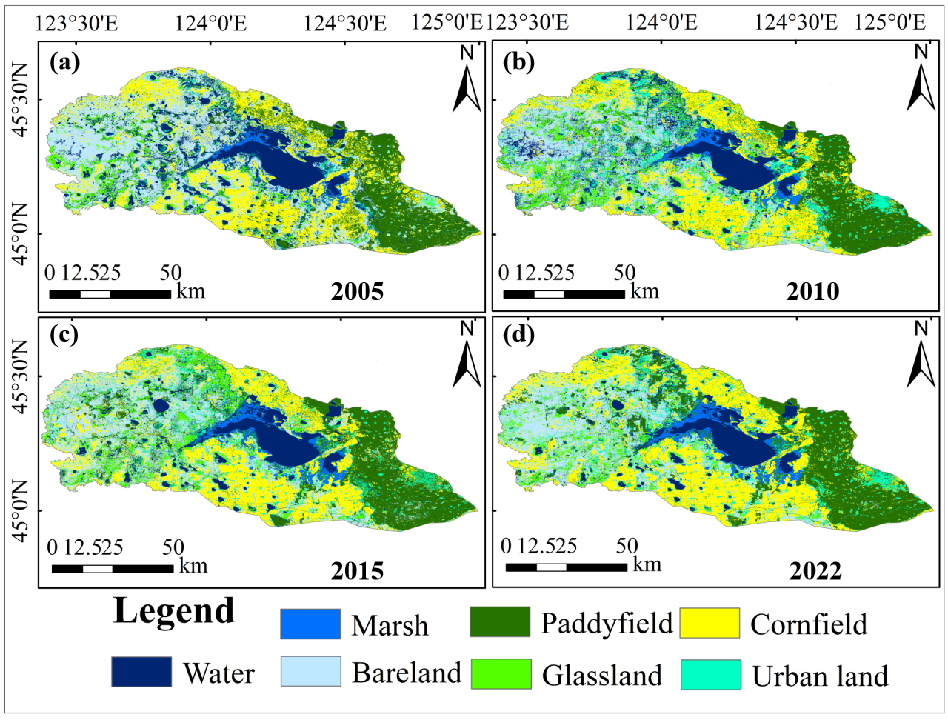


Figure S2. Seven land cover types (including marsh, paddyfield, cornfield, water, bareland, glassland, and urban land) changes in Chagan Lake catchment between 2005 and 2022.


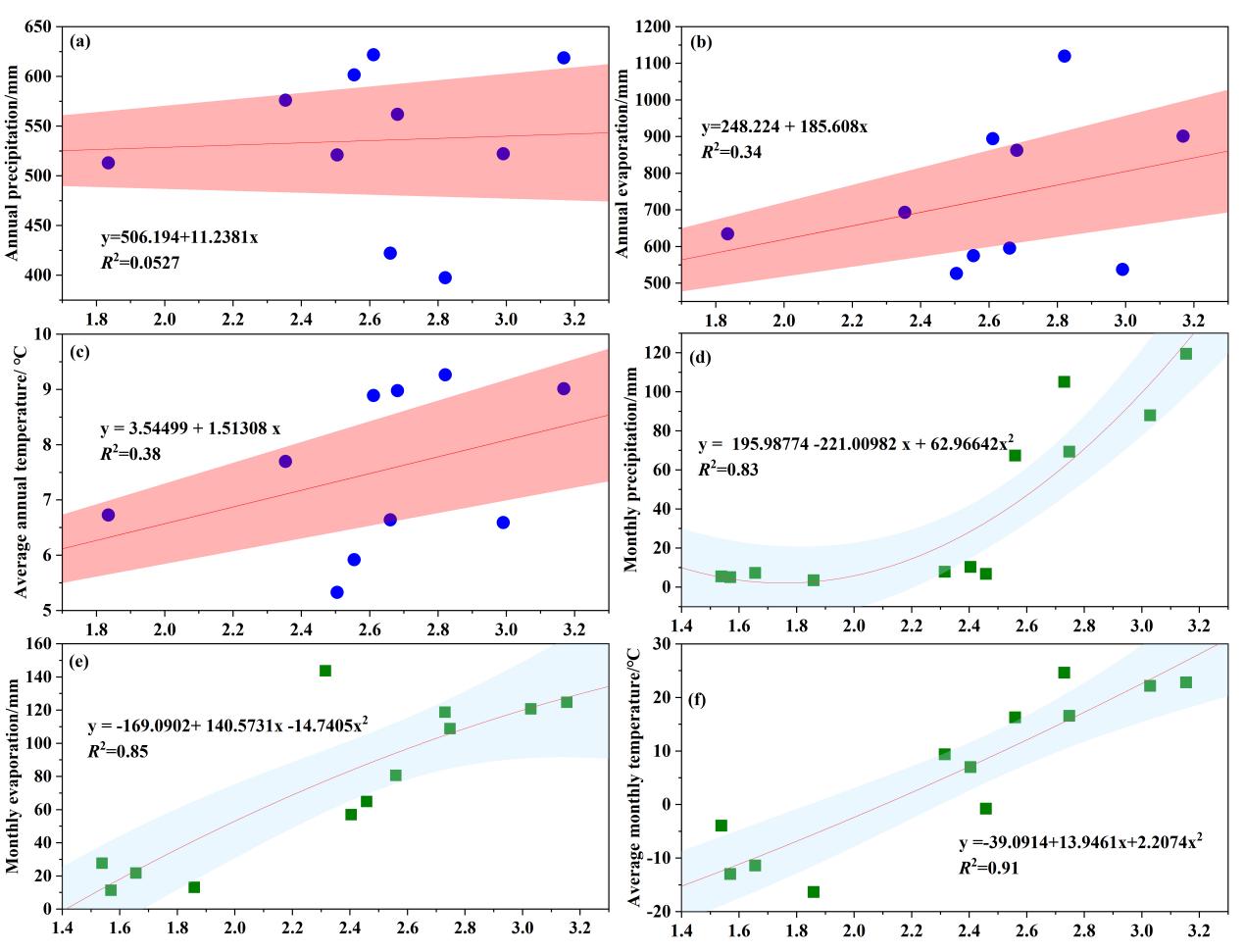


Figure S3. Relationship between meteorological elements and waterbird diversity.


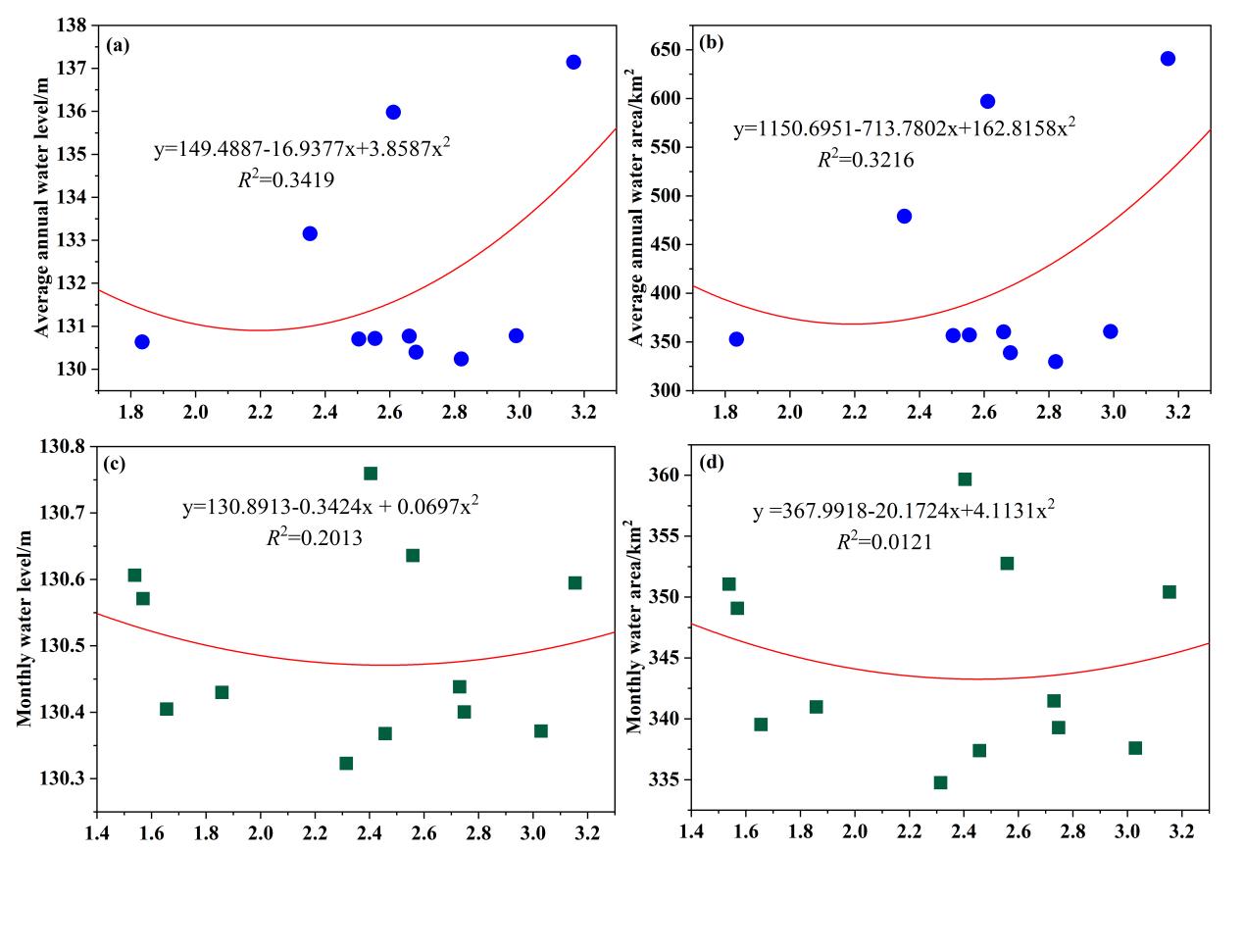


Figure S4 Relationship between hydrologic elements and waterbird diversity.

Figure S5 Relationship between water quality elements and waterbird diversity.


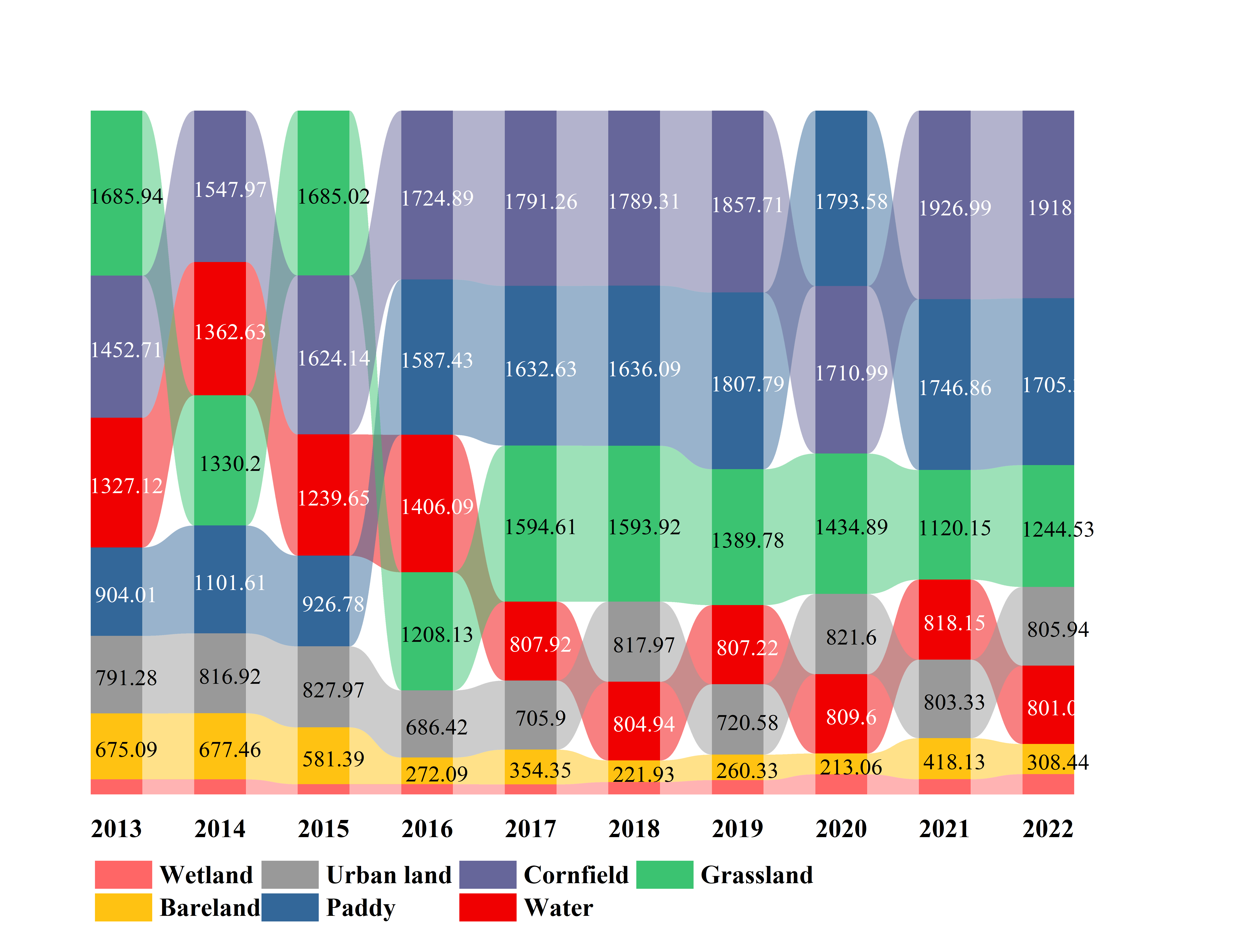


Figure S6 Annual changes in waterbird populations under different land use types.
