# Supplementary material for: Mechanism of Waterbird Diversity Succession and Its Contribution to Nutrient Loads in Chagan Lake, China
Source: Ecol Evol. 2025 Dec 9;15(12):e72583. doi: 10.1002/ece3.72583 (PMC12687063; doi:10.1002/ece3.72583)
Supplement: Supplementary file 2 — Table S1: ece372583‐sup‐0002‐TableS1.docx. [file ECE3-15-e72583-s001.docx]

**Table S1 The list of order, family, species, IUCN standard, pretection level and habtats of birds in Chagan Lake Wetland**

| N | Order | Family | Species | IUCN  Standard | Protection level | Habitats | | | | | |
| --- | --- | --- | --- | --- | --- | --- | --- | --- | --- | --- | --- |
|  |  |  |  |  |  | Open water | Marsh wetland | Shoreside | Forest grassland | Cropland | Sky |
| 1 | Columbiformes | Columbidae | *Streptopelia decaocto* | LC | None | - | - | - | ＋ | ＋ | ＋ |
| 2 |  |  | *Columba rupestris* | LC |  | - | - | - | - | - | ＋ |
| 3 |  |  | *Columba livia domestica* | LC |  | - | - | - | - | ＋ | - |
| 4 |  |  | *Streptopelia orientalis* | LC |  | - | - | ＋ | ＋ | ＋ | ＋ |
| 5 |  |  | *Columba livia* | LC |  |  | ＋ | ＋ | ＋ | - | ＋ |
| 6 |  | Pteroclidae | *Syrrhaptes paradoxus* | LC |  | - | - | - | - | ＋ | - |
| 7 | Galliformes | Phasianidae | *Phasianus colchicus* | LC | "three kinds of" wild animals | ＋ | ＋ | ＋ | ＋ | ＋ | ＋ |
| 8 |  |  | *Coturnix japonica* | NT | "three kinds of" wild animals | - | - | - | - | ＋ | - |
| 9 | Piciformes | Picidae | *Dendrocopos minor* | LC | None | - | - | ＋ | ＋ | ＋ | ＋ |
| 10 |  |  | *Yungipicus canicapillus* | LC |  | - | - | - | ＋ | ＋ | - |
| 11 |  |  | *Picus canus* | LC |  | - | - | - | ＋ | ＋ | - |
| 12 | Passeriformes | Corvidae | *Pica pica* | LC |  | ＋ | - | - | ＋ | ＋ | ＋ |
| 13 |  |  | *Corvus corone* | LC |  | - | - | - | - | - | ＋ |
| 14 |  |  | *Coloeus dauuricus* | LC |  | - | ＋ | ＋ | ＋ | ＋ | - |
| 15 |  |  | *Corvus macrorhynchos* | LC |  | - | - | ＋ | ＋ | ＋ | - |
| 16 |  |  | *Corvus frugilegus* | LC |  | - | ＋ | - | - | - | - |
| 17 |  | Passeridae | *Passer* | LC |  | ＋ | - | - | ＋ | ＋ | ＋ |
| 18 |  |  | *Spinus spinus* | LC |  | - | - | - | ＋ | ＋ | ＋ |
| 19 |  |  | *Passer domesticus* | LC |  | - | ＋ | - | - | ＋ | ＋ |
| 20 |  | Paridae | *Parus major* | LC | "three kinds of" wild animals | - | - | - | ＋ | - | - |
| 21 |  |  | *Poecile palustris* | LC | None | - | - | - | ＋ | ＋ | - |
| 22 |  |  | *Remiz consobrinus* | LC |  | - | - | - | ＋ | - | - |
| 23 |  | Motacillidae | *Motacilla alba* | LC |  | - | - | - | ＋ | - | ＋ |
| 24 |  |  | *Anthus hodgsoni* | LC |  | - | - | - | - | ＋ |  |
| 25 |  |  | *Motacilla tschutschensis Gmelin* | LC |  | - | - | - | ＋ | - | ＋ |
| 26 |  |  | *Anthus richardi* | LC |  | - | - | - | ＋ | - | ＋ |
| 27 |  |  | *Anthus richardi* | LC |  | - | - | - | ＋ | - | ＋ |
| 28 |  | Hirundinidae | *Hirundo rustica* | LC |  | - | - | - | ＋ | - | ＋ |
| 29 |  |  | *Cecropis daurica* | LC |  | - | - | - | - | - | ＋ |
| 30 |  |  | *Hirundo rustica* | LC |  | - | - | - | - | - | ＋ |
| 31 |  |  | *Riparia riparia* | LC |  | - | - | - | - | - | ＋ |
| 32 |  |  | *Acanthis flammea* | LC |  | - | - | - | ＋ | - | - |
| 33 |  | Alaudidae | *Alauda arvensis* | LC | Ⅱ-type of China |  |  |  | ＋ | ＋ | ＋ |
| 34 |  |  | *Melanocorypha mongolica* | LC | Ⅱ-type of China | - | - | - | - | - | ＋ |
| 35 |  |  | *Calandrella brachydactyla* | LC | None | - | - | - | ＋ | ＋ | ＋ |
| 36 |  | Laniidae | *Lanius collurio* | LC |  | - | - | - | ＋ | - | ＋ |
| 37 |  |  | *Lanius cristatus* | LC |  | - | - | - | ＋ | - | ＋ |
| 38 |  |  | *Lanius sphenocercus* | LC |  | - | - | - | - | ＋ | - |
| 39 |  |  | *Lanius tephronotus* | LC |  | - | - | - | ＋ | - | - |
| 40 |  |  | *Lanius excubitor* | LC | "three kinds of" wild animals | - | - | - | ＋ | - | ＋ |
| 41 |  |  | *Lanius excubitor* | LC | "three kinds of" wild animals | - | - | ＋ | - | - | - |
| 42 |  | Emberizidae | *Emberiza cioides cioides* | LC | None | - | - | - | ＋ | ＋ | - |
| 43 |  |  | *Emberiza pusilla* | LC |  | - | - | - | ＋ | - | ＋ |
| 44 |  |  | *Emberiza rustica* | VU |  | - | - | - | - | ＋ | - |
| 45 |  |  | *Emberiza pallasi* | LC |  | - | - | - | ＋ | - | - |
| 46 |  |  | *Emberiza pusilla* | LC |  | - | - | - | ＋ | - | - |
| 47 |  |  | *Emberiza rustica* | VU |  | - | - | - | - | ＋ | ＋ |
| 48 |  |  | *Emberiza schoeniclus* | LC |  | ＋ | - | - | - | - | - |
| 49 |  |  | *Emberiza schoeniclus* | LC |  | - | ＋ | - | - | - | - |
| 50 |  | Sturnidae | *Spodiopsar cineraceus* | LC |  | - | - | - | ＋ | ＋ | - |
| 51 |  |  | *Gracula religiosa* | LC |  | - | - | - | ＋ | ＋ | - |
| 52 |  |  | *Sturnus vulgaris* | LC |  | - | - | - | ＋ | - | ＋ |
| 53 |  | Muscicapidae | *Luscinia svecica* | LC | Ⅱ-type of China | - | - | - | ＋ | - | - |
| 54 |  |  | *Saxicola torquata* | LC | None | - | - | - | ＋ | - | ＋ |
| 55 |  |  | *Tarsiger cyanurus* | LC |  | - | - | - | - | ＋ | - |
| 56 |  |  | *Narcissina Flycatcher* | LC |  | - | - | - | ＋ | - | ＋ |
| 57 |  |  | *Panurus biarmicus* | LC |  | - | - | - | ＋ | ＋ | - |
| 58 |  |  | *Paradoxornis heudei heudei* | NT |  | - | ＋ | - | - | - | - |
| 59 |  | Sylviidae | *Phylloscopus inornatus* | LC |  | - | - | - | ＋ | - | ＋ |
| 60 |  |  | *Phylloscopus fuscatus* | LC |  | - | - | - | ＋ | - | ＋ |
| 61 |  |  | *Acrocephalus orientalis* | LC |  | - | - | - | ＋ | - | - |
| 62 |  |  | *Phylloscopus borealis* | LC |  | - | - | - | - | - | ＋ |
| 63 |  |  | *Acrocephalus bistrigiceps* | LC |  | - | - | - | - | - | ＋ |
| 64 |  |  | *Locustella certhiola* | LC |  | - | - | - | - | - | ＋ |
| 65 |  |  | *Phylloscopus borealis* | LC |  | - | - | - | - | - | ＋ |
| 66 |  | Turdidae | *Turdus naumanni* | LC |  | - | - | - | - | ＋ | - |
| 67 |  | Leiothrichidae | *Garrulax canorus* | LC |  | - | - | ＋ | - | - | - |
| 68 |  | Oriolidae | *Oriolus chinensis* | LC |  | - | - | - | - | ＋ | - |
| 69 |  | Zosteropidae | *Zosterops erythropleurus* | LC | Ⅱ-type of China | - | - | - | ＋ | ＋ | - |
| 70 | Falconiformes | Falconidae | *Falco amurensis* | LC | Ⅱ-type of China | - | - | - | - | ＋ | ＋ |
| 71 |  |  | *Falco tinnunculus* | LC | Ⅱ-type of China | - | - | - | - | ＋ | ＋ |
| 72 |  |  | *Falco peregrinus* | LC | Ⅱ-type of China | - | - | - | - | - | ＋ |
| 73 |  |  | *Falco subbuteo* | LC | Ⅱ-type of China | - | - | - | ＋ | - | - |
| 74 |  |  | *Falco cherrug* | EN | Ⅰ-type of China | - | - | - | ＋ | ＋ | ＋ |
| 75 |  | Accipitridae | *Buteo lagopus* | LC | Ⅱ-type of China | - | - | - | - | - | ＋ |
| 76 |  |  | *Aquila clanga Pallas* | VU | Ⅰ-type of China | - | - | - | - | - | ＋ |
| 77 |  |  | *Circus cyaneus* | LC | Ⅱ-type of China | ＋ | - | - | - | - | - |
| 78 |  |  | *Circus melanoleucos* | LC | Ⅱ-type of China | - | - | - | ＋ | - | ＋ |
| 79 |  |  | *Aquila nipalensis* | EN | Ⅰ-type of China | - | - | - | - | - | ＋ |
| 80 | Strigiformes | Strigidae | *Athene noctua* | LC | Ⅱ-type of China | - | - | - | ＋ | - | ＋ |
| 81 |  |  | *Asio flammeus* | LC | Ⅱ-type of China | - | - | - | ＋ | - | ＋ |
| 82 |  |  | *Bubo scandiacus* | VU | Ⅱ-type of China | - | - | ＋ | ＋ | ＋ | ＋ |
| 83 |  |  | *Asio flammeus* | LC | Ⅱ-type of China | - | - | - | ＋ | - | ＋ |
| 84 | accipitriformes | accipiter | *Accipiter nisus* | LC | Ⅱ-type of China | - | - | - | - | ＋ | ＋ |
| 85 |  |  | *Buteo japonicus* | LC | Ⅱ-type of China | - | - | - | - | - | ＋ |
| 86 |  |  | *Buteo hemilasius* | LC | Ⅱ-type of China | - | - | - | ＋ | ＋ | ＋ |
| 87 |  |  | *Circus spilonotus* | LC | Ⅱ-type of China | - | - | - | - | ＋ | ＋ |
| 88 |  |  | *Haliaeetus albicilla* | LC | Ⅰ-type of China | ＋ | - | - | - | - | - |
| 89 | Anseriformes | Anatidae | *Anser fabalis* | LC | IUCN | ＋ | - | - | - | ＋ | ＋ |
| 90 |  |  | *Anser albifrons* | LC | Ⅱ-type of China | - | - | - | - | ＋ | ＋ |
| 91 |  |  | *Anas crecca* | LC | None | ＋ | - | - | - | - | - |
| 92 |  |  | *Sibirionetta formosa* | LC | Ⅱ-type of China | ＋ | - | - | - | - | - |
| 93 |  |  | *Tadorna ferruginea* | LC | None | ＋ | - | - | - | - | - |
| 94 |  |  | *Anas platyrhynchos* | LC | "three kinds of" wild animals | ＋ | - | - | - | - | - |
| 95 |  |  | *Aix galericulata* | LC | Ⅱ-type of China | ＋ | - | - | - | - | - |
| 96 |  |  | *Bucephala clangula* | LC | None | ＋ | - | - | - | - | - |
| 97 |  |  | *Aythya ferina* | VU |  | ＋ | - | - | - | - | - |
| 98 |  |  | *Bucephala clangula* | LC |  | ＋ | - | ＋ | - | - | ＋ |
| 99 |  |  | *Anas penelope* | LC |  | ＋ | - | ＋ | - | - | ＋ |
| 100 |  |  | *Cygnus* | LC | Ⅱ-type of China | ＋ | - | - | - | - | - |
| 101 |  |  | *Clangula hyemalis* | VU | None | ＋ | - | - | - | - | - |
| 102 |  |  | *Aythya nyroca* | NT |  | ＋ | - | ＋ | - | - | ＋ |
| 103 |  |  | *Melanitta nigra* | LC |  | ＋ | - | ＋ | - | - | ＋ |
| 104 |  |  | *Melanitta fusca* | VU |  | ＋ | - | - | - | - | ＋ |
| 105 |  |  | *Cygnus* | LC | Ⅱ-type of China | ＋ | - | - | - | - | ＋ |
| 106 |  |  | *Aythya ferina* | VU | None | ＋ | - | ＋ | - | - | ＋ |
| 107 |  |  | *Spatula querquedula* | LC |  | ＋ | - | ＋ | - | - | ＋ |
| 108 |  |  | *Anas acuta* | LC |  | ＋ | - | - | - | ＋ | - |
| 109 |  |  | *Mergellus albellus* | LC | Ⅱ-type of China | ＋ | - | ＋ | - | ＋ | - |
| 110 |  |  | *Mergus merganser* | LC | None | ＋ | - | ＋ | - | - | - |
| 111 |  |  | *Anas falcata* | NT |  | ＋ | - | - | - | - | - |
| 112 |  |  | *Tadorna tadorna* | LC |  | ＋ | - | - | - | - | - |
| 113 |  |  | *Anas strepera* | LC |  | ＋ | - | - | - | - | ＋ |
| 114 |  |  | *Tadorna tadorna* | LC |  | ＋ | - | - | - | - | - |
| 115 |  |  | *Aythya nyroca* | NT |  | ＋ | - | - | - | - | - |
| 116 |  |  | *Anas clypeata* | LC | "three kinds of" wild animals | ＋ | - | - | - | - | ＋ |
| 117 |  |  | *Aythya fuligula* | LC | "three kinds of" wild animals | ＋ | - | - | - | - | - |
| 118 |  |  | *Aythya ferina* | VU | None | ＋ | - | - | - | - | - |
| 119 |  |  | *Anas clypeata* | LC | "three kinds of" wild animals | ＋ | - | - | - | - | ＋ |
| 120 |  |  | *Aythya baeri* | CR | Ⅰ-type of China | ＋ | - | - | - | - | ＋ |
| 121 |  |  | *Aythya valisineria* | LC | None | ＋ | - | - | - | - | - |
| 122 |  |  | *Cygnus melanocoryphus* | LC | CITES 2019-Ⅱ | ＋ | - | - | - | - | - |
| 123 |  |  | *Anser anser* | LC | None | - | - | - | - | ＋ | ＋ |
| 124 |  |  | *Anser cygnoides* | EN | Ⅱ-type of China | - | - | - | - | - | ＋ |
| 125 |  |  | *Cygnus cygnus* | LC | Ⅱ-type of China | ＋ | - | - | - | - | - |
| 126 |  |  | *Cygnus olor* | LC | Ⅱ-type of China | ＋ | - | - | - | - | - |
| 127 |  |  | *Cygnus columbianus* | LC | Ⅱ-type of China | ＋ | - | - | - | - | - |
| 128 | Ciconiiformes | Ardeidae | *Botaurus stellaris* | LC | None | - | - | - | - | ＋ | ＋ |
| 129 |  |  | *Ciconia boyciana* | EN | Ⅰ-type of China | ＋ | ＋ | ＋ | ＋ | ＋ | ＋ |
| 130 |  |  | *Ardea purpurea* | LC | None | ＋ | ＋ | ＋ | - | ＋ | ＋ |
| 131 |  |  | *Egretta eulophotes* | VU | Ⅰ-type of China | ＋ | ＋ | - | - | ＋ | - |
| 132 |  |  | *Egretta garzetta* | LC | None | ＋ | ＋ | - | - | ＋ | - |
| 133 | Gruiformes | Rallidae | *Fulica atra* | LC |  | ＋ | ＋ | ＋ | ＋ | ＋ | ＋ |
| 134 |  |  | *Gallinula chloropus* | LC |  | ＋ | - | - | - | - | - |
| 135 |  |  | *Porzana pusilla* | LC |  | - | - | ＋ | - | - | - |
| 136 |  | Gruidae | *Grus grus* | LC | Ⅱ-type of China | - | ＋ | ＋ | ＋ | ＋ | ＋ |
| 137 |  |  | *Grus vipio* | VU | Ⅰ-type of China | - | - | - | - | ＋ | - |
| 138 |  |  | *Grus monacha* | VU | Ⅰ-type of China | - | - | - | - | ＋ | ＋ |
| 139 |  |  | *Grus leucogeranus* | CR | None | - | - | - | - | ＋ | - |
| 140 | Charadriiformes | Charadriidae | *Vanellus vanellus* | NT |  | ＋ | - | ＋ | ＋ | ＋ | ＋ |
| 141 |  |  | *Charadrius dubius* | LC |  | - | - | - | ＋ | - | ＋ |
| 142 |  |  | *Charadrius dubius* | LC |  | - | - | - | ＋ | - | ＋ |
| 143 |  |  | *Pluvialis fulva* | LC |  | - | - | - | - | ＋ | - |
| 144 |  | Laridae | *Larus ridibundus* | LC | "three kinds of" wild animals | ＋ | - | - | - | ＋ | ＋ |
| 145 |  |  | *Larus canus canus* | LC | None | ＋ | - | - | - | - | ＋ |
| 146 |  |  | *Larus relictus* | VU | Ⅰ-type of China | ＋ | - | - | - | - | - |
| 147 |  |  | *Larus vegae* | LC | None | ＋ | - | - | - | - | ＋ |
| 148 |  |  | *Chlidonias hybrida* | LC | "three kinds of" wild animals | ＋ | - | - | - | - | - |
| 149 |  | Scolopacidae | *Limosa limosa* | NT |  | ＋ | - | - | - | - | - |
| 150 |  |  | *Tringa guttifer* | EN | Ⅰ-type of China | ＋ | - | - | - | - | - |
| 151 |  |  | *Limosa limosa* | NT | None |  |  |  | ＋ |  |  |
| 152 |  |  | *Gallinago gallinago* | LC |  | ＋ | - | - | - | - | - |
| 153 |  |  | *Limnodromus scolopaceus* | LC |  | - | ＋ | - | - | - | - |
| 154 |  |  | *Numenius madagascariensis* | EN | Ⅱ-type of China | - | ＋ | ＋ | - | - | - |
| 155 |  |  | *Numenius phaeopus* | LC | None | - | ＋ | ＋ | - | - | - |
| 156 |  |  | *Tringa ochropus* | LC |  | - | ＋ | - | - | - | - |
| 157 |  |  | *Tringa glareola* | LC |  | - | ＋ | - | - | - | - |
| 158 |  |  | *Numenius phaeopus* | LC |  | - | ＋ | - | - | ＋ | - |
| 159 |  |  | *Tringa stagnatilis* | LC |  | ＋ | - | - | - | ＋ | - |
| 160 |  |  | *Actitis hypoleucos* | LC |  | - | - | - | ＋ | ＋ | ＋ |
| 161 |  |  | *Tringa erythropus* | LC |  | - | ＋ | ＋ | - | - | - |
| 162 |  |  | *Scolopax rusticola* | LC |  | ＋ | ＋ | - | - | - | - |
| 163 |  |  | *Limosa lapponica* | NT |  | - | ＋ | - | - | - | - |
| 164 |  | Ibidorhynchidae | *Ibidorhyncha struthersii* | LC | Ⅱ-type of China | ＋ | - | - | - | - | ＋ |
| 165 |  | Recurvirostridea | *Himantopus himantopus* | LC | None | ＋ | - | - | - | - | ＋ |
| 166 |  |  | *Recurvirostra avosetta* | LC | "three kinds of" wild animals | ＋ | - | - | ＋ | ＋ | ＋ |
| 167 |  | Trichodactylidae | *Turnix tanki* | LC | None | ＋ | - | - | - | - | - |
| 168 |  | Haematopsidae | *Haematopus ostralegus* | NT |  | - | ＋ | ＋ | - | - | - |
| 169 |  | Glareolidae | *Glareola maldivarum* | LC |  | - | - | - | - | ＋ | - |
| 170 | Suliformes | Phalacrocoracidae | *Phalacrocorax carbo* | LC |  | ＋ | - | - | - | - | - |
| 171 | Cuccliformes | Cuculidae | *Cuculus micropterus* | LC |  | - | - | - | ＋ | - | ＋ |
| 172 |  |  | *Cuculus canorus bakeri* | LC |  | - | - | ＋ | ＋ | ＋ | ＋ |
| 173 | Lariformes | Laridae | *Larus schistisagus* | LC |  | ＋ | - | - | - | - | ＋ |
| 174 |  |  | *Sterna hirundo* | LC |  | ＋ | - | - | - | - | ＋ |
| 175 |  |  | *Larus ichthyaetus* | LC |  | ＋ | - | ＋ | - | - | ＋ |
| 176 |  |  | *Larus argentatus mongolicus* | LC |  | ＋ | ＋ | - | - | - | ＋ |
| 177 |  |  | *Chlidonias leucopterus* | LC |  | ＋ | ＋ | - | - | - | ＋ |
| 178 |  |  | *Gelochelidon nilotica* | LC |  | - | - | - | - | - | ＋ |
| 179 |  |  | *Sterna hirundo* | LC |  | ＋ | - | - | - | ＋ | ＋ |
| 180 | Podiciformes | Podicipedidae | *Podiceps cristatus* | LC |  | ＋ | ＋ | - | - | ＋ | ＋ |
| 181 |  |  | *Podiceps auritus* | VU |  | ＋ | - | - | ＋ | ＋ | ＋ |
| 182 |  |  | *Tachybaptus ruficollis* | LC |  | ＋ | ＋ | - | ＋ | ＋ | ＋ |
| 183 |  |  | *Podiceps auritus* | VU | Ⅱ-type of China | - | - | - | ＋ | - | - |
| 184 | Pelecaniformes | Ardeidae | *Ardea cinerea* | LC | "three kinds of" wild animals | ＋ | ＋ | ＋ | ＋ | ＋ | ＋ |
| 185 |  |  | *Platalea leucorodia* | LC | CITES 2019-Ⅱ | ＋ | ＋ | ＋ | ＋ | ＋ | ＋ |
| 186 |  |  | *Ardea garzetta* | LC | Ⅰ-type of China | ＋ | ＋ | ＋ | - | ＋ | ＋ |
| 187 |  |  | *Ardea alba* | LC | None | ＋ | - | ＋ | - | - | - |
| 188 |  |  | *Ixobrychus sinensis* | LC |  | - | ＋ | - | - | - | ＋ |
| 189 |  |  | *Ixobrychus eurhythmus* | LC |  | - | - | - | ＋ | - | - |
| 190 |  |  | *Ixobrychus eurhythmus* | LC |  | ＋ | ＋ | - | - | - | - |
| 191 |  | Threskiorothidae | *Platalea minor* | EN | Ⅰ-type of China | - | ＋ | - | - | - | - |
| 192 | Bucerotiformes | Upupidae | *Upupa epops* | LC | None | ＋ | ＋ | ＋ | - | ＋ | ＋ |
| 193 | Coraciiformes | Alcedinidae | *Alcedo atthis* | LC |  | - | - | - | - | - | ＋ |
| 194 | Apodiformes | Apodidae | *Apus nipalensis* | LC |  | ＋ | - | - | ＋ | - | - |
